# Risk of osteoarthritis in patients with hidradenitis suppurativa: a global federated health network analysis

**DOI:** 10.3389/fimmu.2023.1285560

**Published:** 2023-12-19

**Authors:** Hui-Chin Chang, Chih-Lung Wu, Tsu-Man Chiu, Wen-Chieh Liao, Shuo-Yan Gau

**Affiliations:** ^1^ Evidence-based Medicine Center, Chung Shan Medical University Hospital, Taichung, Taiwan; ^2^ Library, Chung Shan Medical University Hospital, Taichung, Taiwan; ^3^ School of Medicine, Chung Shan Medical University, Taichung, Taiwan; ^4^ Department of Orthopedic Surgery, Chung Shan Medical University Hospital, Taichung, Taiwan; ^5^ Department of Dermatology, Chung Shan Medical University Hospital, Taichung, Taiwan; ^6^ Institute of Medicine, Chung Shan Medical University, Taichung, Taiwan; ^7^ Department of Post-Baccalaureate Medicine, College of Medicine, National Chung Hsing University, Taichung, Taiwan; ^8^ Doctoral Program in Tissue Engineering and Regenerative Medicine, College of Medicine, National Chung Hsing University, Taichung, Taiwan; ^9^ Institute of Medical Education, Chi Mei Medical Center, Tainan, Taiwan

**Keywords:** hidradenitis suppurativa, osteoarthritis, cohort, epidemiology, electronic medical records

## Abstract

**Background:**

Osteoarthritis and hidradenitis suppurativa (HS) share a common inflammatory pathway. However, whether patients with HS have higher risk developing osteoarthritis remained unclear.

**Methods:**

A retrospective cohort design was adopted in this study. Electronic medical records had been retrieved from the US collaborative network in the TriNetX research network. A propensity score matching of 1:1 was performed to match for covariates. In total, 50,931 patients with HS and the same amount of non-HS controls were identified for analyses. Hazard ratio (HR) of osteoarthritis in patient with HS was calculated.

**Results:**

Risk of patients with HS developing osteoarthritis was 1.37-fold higher than that of non-HS controls [95% confidence interval (CI), 1.21–1.55] when followed up for 1 year. The significance remained when the follow-up periods were extended to 3 years and 5 years. When osteoarthritis was stratified on occurring sites, the HR of knee osteoarthritis was 1.19 (95% CI, 1.09–1.29) and the HR of hip osteoarthritis was 1.17 (95% CI, 1.01–1.35) in the 5-year follow-up. The 5-year risk of osteoarthritis remained significant in sensitivity models.

**Conclusion:**

Patients with HS were of high risk of developing osteoarthritis compared with people without HS. The clinical association was recommended to be considered while approaching patients with HS.

## Introduction

Hidradenitis suppurativa (HS) is a chronic inflammatory skin disease that leads to the dysfunction of keratinocytes and causes pruritus and pain in patients’ skin lesions (1). According to a recent meta-analysis, the worldwide prevalence of HS was estimated to be 0.4% (2). Comorbidities of HS were found to be involved in various organ systems (3–5). Because proinflammatory cytokines play a critical role in the pathogenesis pathway of HS, it has been reported to be associated with many inflammatory diseases, such as rheumatoid arthritis and psoriatic arthritis (6, 7).

Osteoarthritis could have a substantial impact on the quality of life for patients (8), leading to the gradual breakdown of joint cartilage and causing pain, stiffness, and functional impairment (9). Incident osteoarthritis spans across different age groups, affecting both men and women. A recent global analysis indicated that women presented a higher global prevalence of osteoarthritis than men, and the prevalence increased with age (10). Although aging is regarded as one of the critical risk factors for osteoarthritis, this disease is not solely age-dependent. Risk factors, including inflammation status, obesity, and a history of joint injury, play crucial roles in influencing its onset (11, 12).

The association between HS and inflammatory arthritis has been a subject of prolonged discussion (7). HS has been reported to pose more than a three-fold risk of developing inflammatory arthritis compared to non-HS individuals (6). However, to the best of our knowledge, there is a lack of evidence describing the real-world association between HS and osteoarthritis, despite the existence of overlap in immunological pathways between the two diseases (13, 14). Therefore, we conducted a retrospective cohort analysis to provide further information regarding the risk of osteoarthritis in patients with HS.

## Methods and materials

A retrospective cohort design was adopted for this study, with analyses conducted using datasets from the TriNetX research network. TriNetX is a global platform that provides access to electronic medical records from collaborative health care organizations (HCOs), comprising over 120 institutions. The platform is widely used to explore connections between different exposures and their outcomes (15–17).

In this research, our focus was on utilizing the US collaborative network, relying on records from HCOs within the United States. The dataset for extraction included data from 60 HCOs across the nation, comprising a prospectively updating dataset of approximately 88 million patient records for further analysis. Within the TriNetX platform, diseases were characterized using International Classification of Diseases, Tenth Revision, Clinical Modification (ICD-10-CM) codes, and medications were categorized using Anatomical Therapeutic Chemical and RxNorm coding systems. Details of the applied codes in this study are presented in **Table S1**.

**Figure 1:**
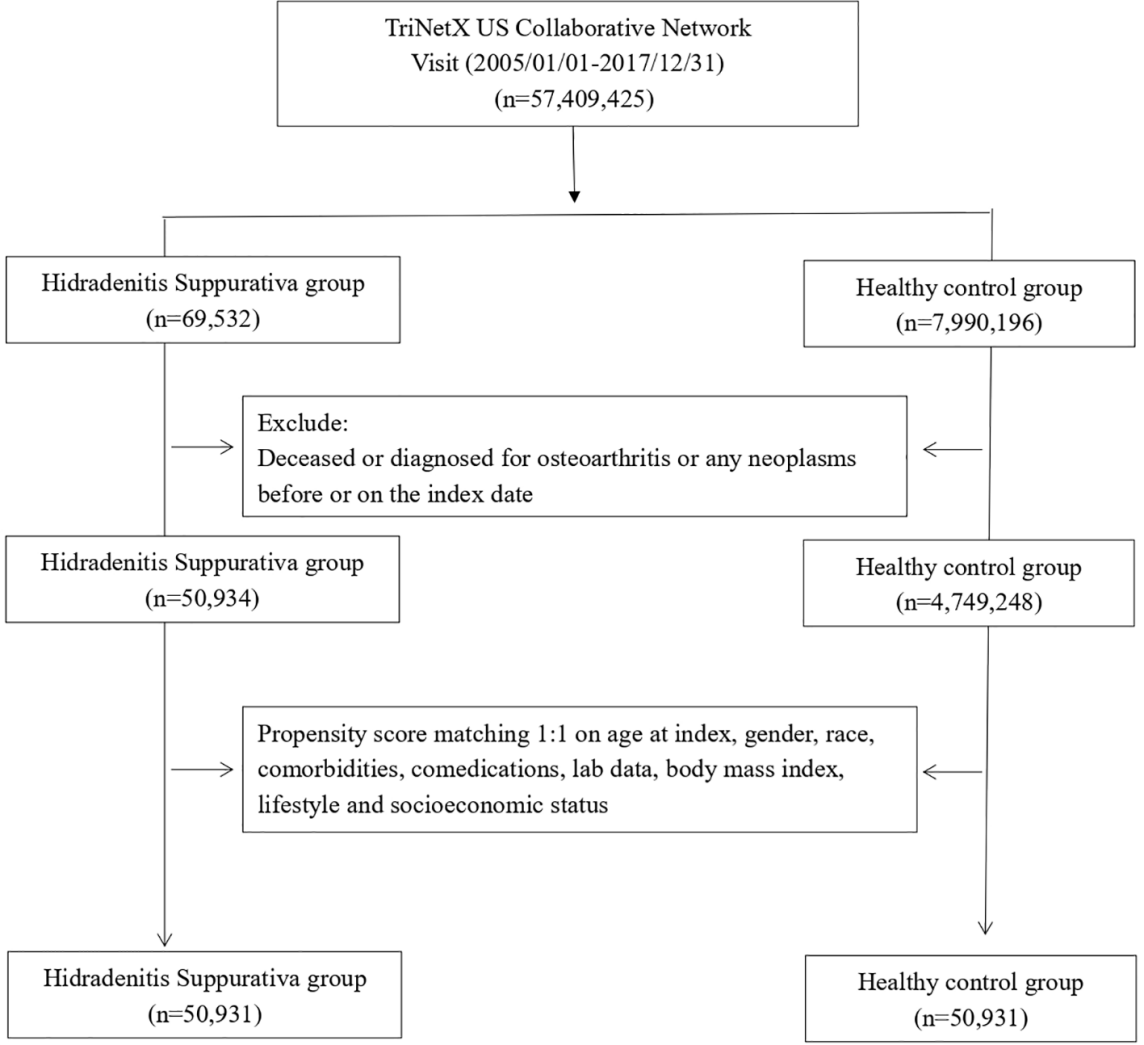
Flowchart of participant selection.

We enrolled patients who had documented visits and received an HS diagnosis within the study time frame, spanning from 1 January 2005 to 31 December 2017. Since TriNetX was prospectively updated, this study period ensured that every included patient underwent a follow-up for a minimum of 5 years. The index date for the HS cohort was set as the diagnosis date of HS. Individuals under 18 years old, those who died before the index date, and those with a history of cancer before the index date were excluded from this study. Healthy controls were identified on the basis of the records of underwent health examinations. To create a proper control group, we implemented propensity score matching. Covariates, including age at index, sex, race, body mass index, status of comorbidities (including diabetes mellitus, hypertension, hyperlipidemia, fracture, and osteoporosis), status of comedication use (glucocorticoids), status of smoking, alcoholism and substance use, medical utilization status, lab data C-reactive protein (CRP), and socioeconomic status (problems related to housing and economic circumstances, and persons with potential health hazards related to socioeconomic and psychosocial circumstances), were set as variables for matching. The end point of this study was defined as incident osteoarthritis. In this study, 1:1 propensity score matching was performed, and 50,323 patients with HS and the same number of non-HS controls were identified for analyses (**Figure 1**).

We calculated the hazard ratio of osteoarthritis in the HS group. In stratification analyses, we assessed the risk of osteoarthritis in different age and sex subgroups. In addition, sensitivity analyses were conducted using diverse analytical models. This included the use of various claim-based algorithms with a high positive predictive value (18, 19), consideration of different matching covariates, and implementation of varying washout periods. These approaches were employed to mitigate potential biases.

The framework of the TriNetX research network system was utilized for statistical analyses in this study, conducted between 20 November 2023 and 22 November 2023. Standardized difference (SD) calculations were performed, and a value exceeding 0.1 indicated a statistically meaningful differentiation between the two compared groups. Propensity score matching was executed using a greedy nearest neighbor matching technique with a specific caliper of 0.1. Hazard ratios (HRs) were assessed, and their corresponding 95% confidence intervals (CIs) were calculated whenever applicable.

### Statement of ethics

“The dataset within the TriNetX research network underwent de-identification following the guidelines outlined in Section §164.514(a) of the HIPAA Privacy Rule. This de-identification process was certified by a qualified expert, as outlined in Section §164.514(b)(1) of the HIPAA Privacy Rule. All analyses conducted in this study did not involve any intervention or direct engagement with human participants. Consequently, studies utilizing the TriNetX research network were deemed exempt from requiring permission from an Institutional Review Board (IRB) (20)”.

## Results

**Table 1** presented the baseline characteristics of participants. Patients in the HS group were 32.8 years old on average. Of the 50,931 patients with HS, 37,612 (73.8%) were women. Before matching, the baseline characteristics of age, sex, and race between the HS group and the control group were significantly different. After matching, baseline characteristics become similar between the two groups.

**Table 1:** Baseline characteristics of study subjects (before and after propensity score matching).

	Before matching			After matching ^a^		
	HS cohort (n = 50,934)	Control cohort (n = 4,749,248)	Std diff	HS cohort (n = 50,931)	Control cohort (n = 50,931)	Std diff
Age at index						
Mean ± SD	32.7 ± 13.7	36.4 ± 20.2	**0.21**	32.7 ± 13.7	33.2 ± 14.2	0.03
Sex						
Male	12,728 (25.0)	2,054,663 (43.3)	0.39	12,728 (25.0)	12,665 (24.9)	0.00
Female	37,615 (73.9)	2,568,755 (54.1)	0.42	37,612 (73.8)	37,621 (73.9)	0.00
Race, n (%)						
White	22,501 (44.2)	2,802,552 (59.0)	0.30	22,501 (44.2)	22,367 (43.9)	0.01
Black or African American	17,715 (34.8)	709,601 (14.9)	0.47	17,712 (34.8)	17,987 (35.3)	0.01
Asian	842 (1.7)	168,173 (3.5)	0.12	842 (1.7)	1,619 (3.2)	0.10
American Indian or Alaska Native	228 (0.4)	15730 (0.3)	0.02	228 (0.4)	183 (0.4)	0.01
Socioeconomic status						
Socioeconomic/psychosocial circumstances problem	959 (1.9)	31,870 (0.7)	0.11	958 (1.9)	852 (1.7)	0.02
Lifestyle						
Alcohol dependence, smoking and substance use	5,590 (11.0)	138,867 (2.9)	0.32	5,587 (11.0)	5,668 (11.1)	0.01
Comorbidities						
Hypertension	5,487 (10.8)	384,694 (8.1)	0.09	5,486 (10.8)	5,509 (10.8)	0.00
Diabetes mellitus	3,301 (6.5)	165,703 (3.5)	0.14	3,299 (6.5)	3,223 (6.3)	0.01
Hyperlipidemia	2,844 (5.6)	250,222 (5.3)	0.01	2,844 (5.6)	2,730 (5.4)	0.01
Osteoporosis	173 (0.3)	30,227 (0.6)	0.04	173 (0.3)	134 (0.3)	0.01
Fracture of shoulder and upper arm	288 (0.6)	23,525 (0.5)	0.01	288 (0.6)	217 (0.4)	0.02
Fracture of femur	90 (0.2)	7,070 (0.1)	0.01	90 (0.2)	67 (0.1)	0.01
Fracture of lower leg	460 (0.9)	24,218 (0.5)	0.05	460 (0.9)	376 (0.7)	0.02
Medications						
Glucocorticoids	9,580 (18.8)	418,016 (8.8)	0.29	9577 (18.8)	9,474 (18.6)	0.01
Medical Utilization Status						
Ambulatory visit	28,403 (55.8)	2140,433 (45.1)	0.22	28,402 (55.8)	28,480 (55.9)	0.00
Inpatient visit	8,032 (15.8)	476,128 (10.0)	0.17	8,032 (15.8)	8,062 (15.8)	0.00
Laboratory data						
BMI, n (%)						
≧ 25 (kg/m^2^)	7,800 (15.3)	329,274 (6.9)	0.27	7,797 (15.3)	7,818 (15.4)	0.00
C reactive protein, n (%)						
≧ 3 (mg/L)	1,778 (3.5)	55,486 (1.2)	0.15	1,775 (3.5)	1,690 (3.3)	0.01

If the patient is less or equal to 10, then the results show the count as 10.

Bold font represents that a standardized difference was more than 0.1.

HS, hidradenitis suppurativa.

a Propensity score matching was performed on age at index, sex, race, body mass index, status of comorbidities (including diabetes mellitus, hypertension, hyperlipidemia, fracture, and osteoporosis), status of comedication use (glucocorticoids), status of smoking, alcoholism and substance use, medical utilization status, lab data (CRP), and social economic status (problems related to housing and economic circumstances, and persons with potential health hazards related to socioeconomic and psychosocial circumstances).

Osteoarthritis risk in patients with HS was presented in **Table 2**. Risk of patients with HS developing osteoarthritis was 1.37-fold higher than that of non-HS controls (95% CI, 1.21–1.55) when followed up for 1 year. The significance remained when the follow-up periods were extended to 3 years (HR, 1.34; 95% CI, 1.25, 1.44) and 5 years (HR, 1.34; 95% CI, 1.27–1.41). When osteoarthritis was stratified on occurring sites, the HR of knee osteoarthritis was 1.19 (95% CI, 1.09–1.29) and the HR of hip osteoarthritis was 1.17 (95% CI, 1.01–1.35) in the 5-year follow-up (**Table 3**). The 5-year risk of osteoarthritis remained significant in sensitivity models applying different matching algorithms (**Table S2**) and different definition of HS based on ICD-10-CM-codes (**Table S3**). In the sensitivity analyses applying various washout periods, significance was also observed (**Table S4**). When excluding the incident osteoarthritis diagnosed within 36 months after index date, the HR for patients with HS having osteoarthritis in future 5 years was 1.31 (95% CI, 1.22–1.41).

**Table 2:** Risk of osteoarthritis under different follow-up time ^a^.

Outcomes	Hazard ratio (95% Confidence interval) ^b^		
	1 year	3 years	5 years
Osteoarthritis	**1.37 (1.21, 1.55)**	**1.34 (1.25, 1.44)**	**1.34 (1.27, 1.41)**

HS, hidradenitis suppurativa.

a Data presented here were the value of follow-up from 90 days after index date to the respective following-up years.

b Propensity score matching was performed on age at index, sex, race, body mass index, status of comorbidities (including diabetes mellitus, hypertension, hyperlipidemia, fracture, and osteoporosis), status of comedication use (glucocorticoids), status of smoking, alcoholism and substance use, medical utilization status, lab data (CRP), and social economic status (problems related to housing and economic circumstances, and persons with potential health hazards related to socioeconomic and psychosocial circumstances).

Bold values indicates that those values were of statistical significance.

**Table 3:** Risk of site of osteoarthritis in the 5-year follow-up ^a^.

Outcomes	Hazard ratio (95% confidence interval) ^b^
Knee osteoarthritis	**1.19 (1.09, 1.29)**
Hip osteoarthritis	**1.17 (1.01, 1.35)**

HS, hidradenitis suppurativa.

a Data presented here were the value of follow-up from 90 days after index date to the respective following-up years.

b Propensity score matching was performed on age at index, sex, race, body mass index, status of comorbidities (including diabetes mellitus, hypertension, hyperlipidemia, fracture, and osteoporosis), status of comedication use (glucocorticoids), status of smoking, alcoholism and substance use, medical utilization status, lab data (CRP), and socioeconomic status (problems related to housing and economic circumstances, and persons with potential health hazards related to socioeconomic and psychosocial circumstances).

Bold values indicates that those values were of statistical significance.

Results of stratification analyses were presented in **Table 4**. The 5-year risk of developing future osteoarthritis in male patients with HS was 1.20-fold higher than that of non-HS male population (95% CI, 1.07–1.33). For female patients with HS, the HR was 1.31 (95% CI, 1.23–1.40). In patients with HS ranged from 18 to 64 years old, the HR of developing osteoarthritis was 1.19 (95% CI, 1.07–1.33), whereas, in patients with HS greater than 65 years old, the HR was 1.38 (95% CI, 1.24–1.54), compared with non-HS controls in respective age subgroups.

**Table 4:** Stratification analysis of osteoarthritis risk in patients with HS.

	Cases occurring new-onset osteoarthritis		
Subgroups	HS cohort (no. of event/HS patient amount in each subgroup)	Control cohort (no. of event/non-HS patient amount in each subgroup)	HR (95% CI) ^a^
Gender			
Male	707/12,376	597/12,376	**1.20 (1.07, 1.33)**
Female	2,084/36,498	1,588/36,498	**1.31 (1.23, 1.40)**
Age at index date			
18–64 years old	745/12,728	625/12,728	**1.19 (1.07, 1.33)**
≥ 65 years old	722/3,950	555/3,950	**1.38 (1.24, 1.54)**

a Propensity score matching was performed on age at index, sex, race, body mass index, status of comorbidities (including diabetes mellitus, hypertension, hyperlipidemia, fracture, and osteoporosis), status of comedication use (glucocorticoids), status of smoking, alcoholism and substance use, medical utilization status, lab data (CRP), and socioeconomic status (problems related to housing and economic circumstances, and persons with potential health hazards related to socioeconomic and psychosocial circumstances).

b In order to protect the privacy of participants, the TriNetX system was not able to present the exact number of participant if the number was less than 10. Hence, for these stratification groups, we were not able to calculate the hazard ratio.

Bold values indicates that those values were of statistical significance.

## Discussion

According to the results of the current study, patients with HS were associated with a higher risk of future osteoarthritis compared to non-HS controls, with an HR of 1.37 (95% CI, 1.21–1.55) within the first year after the index date. This elevated risk remained statistically significant in different sex and age subgroups.

Pathogenesis of HS involved the elevation of proinflammatory cytokines that could trigger subsequent immunological cascades (21). Although the pathogenesis of HS has not been fully understood, it was pointed out that sustained inflammatory reaction within dermatological diseases had the potential to trigger systemic immunological involvement, consequently fostering the emergence of comorbidities in various organ systems (22–24). Given these conditions, the issue of HS comorbidities had long been discussed and was acknowledged as intricate, encompassing several different functional problems including allergic status (25), gastrointestinal dysfunctions (26, 27), and renal diseases (28). Among the comorbidities of HS, integrated pieces of evidence had suggested that spondyloarthritis and rheumatoid arthritis were of high prevalence and incidence in the HS population (6, 29). A recent small-scale case-control study stated that some of the bone function indicators, such as trabecular bone score and hip bone mineral density, were low in patients with HS compared with that in controls (30). Although previous meta-analyses stated that HS could be associated with other inflammatory arthritis (6), the association between HS and osteoarthritis has not been clearly clarified because of insufficient data in observational studies. Thereby, to fulfill the knowledge gap, we provided real-world information regarding the risk of osteoarthritis in patients with HS after considering the influence of various confounders.

Clinically, patients with syndromic HS were often observed to present with articular involvement, and the association between synovitis, acne, pustulosis, hyperostosis, and osteitis (SAPHO) syndrome has long been discussed (31, 32). In the involved pathways of HS and osteoarthritis, some of the involved key cytokines were in common. For instance, in patients with HS, because of the chronic inflammation status in follicles, the proinflammatory cytokines, such as interleukin 1b (IL-1b) and tumor necrosis factor-alpha (TNF-alpha), would be elevated and lead to systemic inflammation (1, 21). In patients with osteoarthritis, these cytokines could also result in the impairment of tissues in the musculoskeletal system, wherein IL-1b could play a critical role in cartilage damage and TNF-alpha was associated with triggering further inflammatory reactions (33). Moreover, the influence of IL-17 was also a possible attribution of the HS–osteoarthritis association. The activation of IL-17 cytokine was regarded as one of the critical process contributing to the dysfunction of keratinocytes and systemic inflammation (14), and the concentration of IL-17 was found to be high in patients with HS (34). IL-17 contributes to the pathogenesis of HS by stimulating the proliferation of keratinocytes. This, in turn, prompts keratinocytes to produce inflammatory mediators such as antimicrobial peptides and cytokines, which could drive inflammation and recruit immune cells (14, 35). Studies revealed that the concentration of IL-17 in patients with osteoarthritis was higher than those without osteoarthritis (36). IL-17 was proposed to play a potential role in the promotion of chondrocyte senescence and apoptosis in patients with osteoarthritis (37). It stimulates the production of reactive oxygen species, chemokines, and inflammatory factors like IL-1β in chondrocytes. This results in premature chondrocyte senescence, cell cycle dysfunction, and apoptosis, contributing to cartilage breakdown (38). Moreover, in the recent *in vitro* and *in vivo* studies, IL-17 was reported to promote the occurrence of cartilage deformation (13, 39, 40). However, in the current study design, we were not able to monitor the patients’ inflammatory status and clarify the actual cytokine reaction pathway. Therefore, future clinical studies were needed to elucidate the role of the aforementioned cytokines in the observed HS–osteoarthritis association.

There is currently a great interest surrounding the emerging systemic therapeutics for HS, including potential combination regimens, which may not only improve HS symptoms but beneficially impact comorbidities like inflammatory arthritis (41). These treatments could not only alleviate HS symptoms but also positively affect related conditions such as inflammatory comorbidities. Clinical trials have demonstrated the effectiveness of anti–IL-17 biologics like secukinumab and bimekizumab in reducing the severity of HS (42, 43). Pieces of evidence also suggest that Janus kinase inhibitors such as baricitinib and tofacitinib could be beneficial in HS management (44). These systemic therapies could potentially reduce chronic inflammation associated with common comorbidities; however, current pieces of evidence regarding the head-to-head comparison evaluating whether biologics use could be effective in reducing the inflammatory comorbidity status in patients with HS are lacking. It is important to carefully monitor the response of related conditions like arthritis to these promising HS treatments.

In our study, we found that the risk of patients with HS developing osteoarthritis was slightly higher within a 1-year follow-up period (HR, 1.37; 95% CI, 1.21–1.55) compared with the 3-year (HR, 1.34; 95% CI, 1.25–1.44) and 5-year (HR, 1.34; 95% CI, 1.27–1.41) follow-up periods. The marginally increased risk of an outcome event in the short-term follow-up period could potentially be due to the effect of reversed causation bias (45), which is common in real-world studies (46). Under the same washout periods, incident osteoarthritis events could inadvertently include those who already have underlying osteoarthritis but are diagnosed shortly after an HS diagnosis. To address this issue of reverse causality, we applied different washout periods in sensitivity models, and the association between HS and osteoarthritis remained significant in each model.

Limitation of this study should be considered while interpreting the results. First, we were only able to provide follow-up time up to 5 years after the index date in the current study design. Hence, we were not able to evaluate the long-term association between HS and osteoarthritis. Second, although we have tried our best to address the confounding bias caused by potential confounders via propensity score matching method, some unknown confounders could still influence the evaluation of association between the two diseases. Third, although we have applied validated coding algorithms in the definition of HS (18, 19), as in other studies utilizing database to perform secondary analyses (47, 48), misclassification bias due to the coding algorithm could still lead to potential imprecise results and should not be neglected while interpreting the clinical implication of the current study.

To recapitulate, we report that patients with HS were of high risk of developing osteoarthritis compared with people without HS, and the risk was 1.41-fold (95% CI, 1.24–1.60) in the first year after HS diagnosis. The clinical association was recommended to be taken into account in the treatment plan while approaching patients with HS.

## Data availability statement

The data analyzed in this study is subject to the following licenses/restrictions: Data in this study were retrieved from TriNetX Research Network. All data available in the database were administrated by the TriNetX platform. Detailed information can be retrieved at the official website of the research network (https://trinetx.com). Requests to access these datasets should be directed to TriNetX platform (https://trinetx.com).

## Ethics statement

Ethical approval was not required for the study involving humans in accordance with the local legislation and institutional requirements. Written informed consent to participate in this study was not required from the participants or the participants’ legal guardians/next of kin in accordance with the national legislation and the institutional requirements.

## Author contributions

HC: Conceptualization, Data curation, Investigation, Project administration, Software, Supervision, Writing – original draft, Writing – review & editing. CW: Conceptualization, Investigation, Supervision, Validation, Writing – original draft, Writing – review & editing. WL: Conceptualization, Data curation, Formal analysis, Funding acquisition, Investigation, Project administration, Validation, Writing – original draft, Writing – review & editing. SG: Conceptualization, Data curation, Formal analysis, Investigation, Methodology, Project administration, Validation, Visualization, Writing – original draft, Writing – review & editing. TC: Conceptualization, Formal analysis, Investigation, Methodology, Supervision, Validation, Writing – original draft, Writing – review & editing.
