# Supplementary material for: Risk of osteoarthritis in patients with hidradenitis suppurativa: a global federated health network analysis
Source: Front Immunol. 2023 Dec 19;14:1285560. doi: 10.3389/fimmu.2023.1285560 (PMC10763244; doi:10.3389/fimmu.2023.1285560)
Supplement: Supplementary file 1 [file Table_1.docx]

**Supplementary Files**

**Supplementary Tables**

**Table S1.** Utilized adminstrative codes in the current study **^a^**

| **Description** | **ICD-10-CM codes** |
| --- | --- |
| Hidradenitis Suppurativa | L73.2 |
| Neoplasms | C00-D49 |
| Osteoarthritis | M15-M19 |
| Diabetes mellitus | E08-E13 |
| Hypertension | I10 |
| Hyperlipidemia | E78.5 |
| Socioeconomic and psychosocial circumstances | Z55-Z65 |
| Substance abuse | F10-F19 |
| Fracture of shoulder and upper arm | S42 |
| Fracture of femur | S72 |
| Fracture of lower leg, including ankle | S82 |
| Osteoporosis | M81 |
| Encounter for general examination | Z00 |
| **Medications** | **ATC codes/RxNorms** |
| Glucocorticoids | ATC code: H02AB |
| Corticosteroids | ATC code: C05AA |
| Systemic corticosteroids | ATC code: H02 |
| *TNF alpha inhibitors* |  |
| Adalimumab | RxNorm: 327361 |
| Infliximab | RxNorm: 191831 |
| Etanercept | RxNorm: 214555 |
| *IL-17 inhibitors* |  |
| Ixekizumab | RxNorm: 1745099 |
| Secukinumab | RxNorm: 1599788 |
| Brodalumab | RxNorm: 1872251 |
| *JAK inhibitors* |  |
| Tofacitinib | RxNorm: 1357536 |
| Baricitinib | RxNorm: 2047232 |
| *Antibiotics* |  |
| Doxycycline | RxNorm: 3640 |
| Minocycline | RxNorm:6980 |
| Erythromycin | RxNorm: 4053 |
| Trimethoprim/Sulfamethoxazole | RxNorm: 10829/10180 |
| Clindamycin | RxNorm: 2582 |
| Rifampin | RxNorm: 9384 |
| **Procedures** | CPT codes |
| Incision and drainage | 10060 |
| Intralesional injection | 1003286 |

^a^ICD-10-CM: International Classification of Diseases, Tenth Revision, Clinical Modification; ATC codes: Anatomical Therapeutic Chemical codes; CPT codes, Current Procedural Terminology codes

**Table S2.** Sensitivity analysis: risk of osteoarthritis in HS patients based on different covariate matching models, with 5-year follow up

| Models | Hazard ratio (95% Confidence interval) |
| --- | --- |
| Crude | **1.15 (1.11,1.19)** |
| Model 1^a^ | **1.83 (1.73,1.95)** |
| Model 2^b^ | **1.65 (1.56,1.75)** |
| Model 3^c^ | **1.50 (1.41,1.58)** |
| Model 4^d^ | **1.64 (1.54,1.73)** |
| Model 5^e^ | **1.30 (1.23,1.37)** |
| Model 6^f^ | **1.33 (1.26,1.41)** |

^a^ Propensity score matching was performed on age at index and sex

^b^ Propensity score matching was performed on age at index, sex, race, medical utilization status and socioeconomic status

^c^ Propensity score matching was performed on age at index, sex, race, comorbidities and comedications (glucocorticoids)

^d^ Propensity score matching was performed on age at index, sex, race and overweight status (BMI ≥ 25)

^e^ Propensity score matching was performed on age at index, sex, race, body mass index, comorbidities, comedications status of smoking, alcoholism and substance use, medical utilization status, lab data, socioeconomic status and obesity status (BMI ≥ 30)

^f^ Propensity score matching was performed on age at index, sex, race, body mass index, comorbidities, comedications status of smoking, alcoholism and substance use, medical utilization status, lab data, socioeconomic status and obesity status (BMI ≥ 35)

**Table S3.** Sensitivity analysis: risk of osteoarthritis in HS patients based on different claim-based algorithms with 5-year follow up

| Models | Hazard ratio (95% Confidence interval)^a^ |
| --- | --- |
|  |  |
| Model 1 ^b^ | **1.32 (1.24,1.40)** |
| Model 2 ^c^ | **1.51 (1.42,1.61)** |
| Model 3 ^d^ | **1.63 (1.28,2.08)** |
| Model 4 ^e^ | **1.58 (1.39,1.78)** |
| Model 5 ^f^ | **1.36 (1.27,1.45)** |
| Model 6 ^g^ | **1.43 (1.15,1.79)** |

HS: hidradenitis suppurativa

^a^ Propensity score matching was performed on age at index, sex, race, body mass index, status of comorbidities, status of comedication use, status of smoking, alcoholism and substance use, medical utilization status, lab data and socioeconomic status.

^b^ Only patients diagnosed HS and was never diagnosed of cutaneous abscess by specialists at the same time were included as HS group in this model. The algorithm has been validated in a previous validation study with the PPV of 88%.

^c^ Only patients diagnosed HS and with the record of being prescribed of systemic corticosteroids were included as HS group in this model.

^d^ Only patients diagnosed HS and with the record of being prescribed of TNF alpha inhibitors, other biologics (such as IL-17 inhibitors), or small molecules drugs (such as JAK inhibitors) were included as HS group in this model.

^e^ Only patients diagnosed HS and with the record of underwent drainage and incision were included as HS group in this model.

^f^ Only patients diagnosed HS and with a record of use of antibiotics with anti-inflammatory properties were included as HS group in this model.

^g^ Only patients diagnosed HS and with a record of being prescribed corticosteroids and underwent intralesional injection were included as HS group in this model.

**Table S4.** Sensitivity analysis: risk of osteoarthritis with different wash-out periods ^a^

| Outcomes | Hazard ratio (95% Confidence interval)^b^ | | |
| --- | --- | --- | --- |
|  | 6 months | 12 months | 36 months |
| Osteoarthritis | **1.34 (1.27,1.42)** | **1.35 (1.28,1.43)** | **1.31 (1.22,1.41)** |

^a^ Incident events occurred within each wash-out period were excluded in the corresponding analysis, following up for 5 years after index date

^b^ Propensity score matching was performed on age at index, sex, race, body mass index, status of comorbidities (including diabetes mellitus, hypertension, hyperlipidemia, fracture, osteoporosis), status of comedication use (glucocorticoids), status of smoking, alcoholism and substance use, medical utilization status, lab data (CRP) and social economic status (problems related to housing and economic circumstances, persons with potential health hazards related to socioeconomic and psychosocial circumstances).
